# Supplementary material for: Pattern of Regulatory T Cells, Resident Memory T Cells, and Exhausted T Cells in Human Pericardial Fluid Samples of Cardiovascular Patients
Source: Int J Mol Sci. 2025 Oct 10;26(20):9852. doi: 10.3390/ijms26209852 (PMC12562597; doi:10.3390/ijms26209852)
Supplement: Supplementary file 1 [file ijms-26-09852-s001.zip › ijms-3859657-supplementary.pdf]

Table S1. Difference between recipient T-cell levels and primer graft dysfunction

	PGD		No PGD		p value	q value
	Median	IQR (25-75)	Median	IQR (25-75)		
Treg CD4+	29.60	16.90 - -	34.10	24.45 - 36.30	0.364	0.994
Treg CD4+ FOXP3+	7.24	2.20 - -	7.25	3.38 - 11.34	0.8	0.994
Treg CD4+ FOXP3+ CCR5+	95.90	85.80 - -	82.3	69.75 - 97.40	0.364	0.994
Treg CD4+ FOXP3+ CCR5+ Q1: CCR4- , CXCR3+	0	0 - -	0	0 - 0.07	0.8	0.994
Treg CD4+ FOXP3+ CCR5+ Q2: CCR4+ , CXCR3+	16.90	4.11 - -	17.70	4.82 - 38.20	1	1
Treg CD4+ FOXP3+ CCR5+ Q3: CCR4+ , CXCR3-	78.50	27.20 - -	63.00	48.20 - 78.20	0.8	0.994
Treg CD4+ FOXP3+ CCR5+ Q4: CCR4- , CXCR3-	1.79	0.75 - -	0.51	0.19 - 31.70	0.704	0.994
Treg CD4+ FOXP3+ CCR5-	04.07	1.48 - -	17.70	2.61 - 30.25	0.364	0.994
Treg CD4+ FOXP3+ CD45RA+	6.64	3.53 - -	9.93	3.49 - 26.65	0.439	0.994
Trm CD8+	26.30	16.00 - -	23.10	16.10 - 31.98	0.768	0.994
Trm TCD8+/CCR5+ CXCR3+	17.50	11.00 - -	4.915	2.46 - 30.53	0.197	0.994
Trm CD8+/CD69+ CD103+	14.70	5.87 - -	17.30	12.33 - 27.03	0.509	0.994
Trm CD8+/CD69+ CD103+/CCR5+ CXCR3+	19.00	8.76 - -	3.98	1.73 - 37.10	0.244	0.994
Trm CD8+/CD69+ CD103+/CD49a+	47.10	25.50 - -	40.65	30.53 - 64.53	0.859	0.994
Tex CD4+	25.70	23.60 - -	28.40	20.63 - 39.98	0.676	0.994
Tex CD4+/CCR5+	90.30	34.40 - -	82.30	50.78 - 91.65	0.768	0.994
Tex CD4+/CCR5+/CXCR3+	16.90	16.50 - -	20.70	13.50 - 34.40	0.768	0.994
Tex CD4+/CCR5+/CXCR3+/PD-1+	51.60	6.80 - -	34.95	12.43 - 65.53	0.859	0.994
Tex CD4+ /CCR5+/CXCR3+/PD-1+/TIM-3+	8.33	0.72 - -	5.26	03.02 - 7.67	0.676	0.994
Tex CD4+ /CCR5+/CXCR3+/TIM-3+	8.36	0.99 - -	5.88	2.99 - 10.47	1	1
Tex CD4+ /CCR5-	9.69	3.79 - -	17.70	8.36 - 49.23	0.768	0.994
Tex CD4+ /CCR5-/CXCR3-	60.40	14.00 - -	67.15	43.98 - 94.65	0.591	0.994
Tex CD4+ /CCR5-/CXCR3-/PD-1+	8.54	7.60 - -	9.01	5.36 - 27.05	0.591	0.994
Tex CD4+/CCR5-/CXCR3-/TIM-3+	1.18	0.76 - -	2.53	0.94 - 7.67	0.676	0.994
Tex CD8+	26.40	15.90 - -	25.20	16.45 - 31.85	0.768	0.994
TexCD8+/CCR5+	95.40	82.20 - -	95.40	80.33 - 98.35	1	1
Tex CD8+/CCR5+/CXCR3+	15.30	14.60 - -	13.10	8.54 - 34.23	0.591	0.994
Tex CD8+/CCR5+/CXCR3+/PD-1+	50.60	2.35 - -	40.35	11.33 - 74.885	0.859	0.994
Tex CD8+/CCR5+/CXCR3+/PD-1+/TIM-3+	1.90	1.00 - -	2.79	1.27 - 3.98	0.859	0.994

Tex CD8+/CCR5+/CXCR3+/TIM-3+	01.04	0.54	-	-	1.72	0.79	-	03.05	0.432	0.994
Tex CD8+/CCR5-	4.57	1.29	-	-	4.59	1.69	-	19.68	0.953	0.999
TexCD8+/CCR5-/CXCR3-	36.40	11.90	-	-	90.20	33.80	-	97.28	0.432	0.994
Tex CD8+/CCR5-/CXCR3-/PD-1+	4.11	0.29	-	-	3.46	2.23	-	7.36	1	1
TexCD8+/CCR5-/CXCR3-/TIM-3+	4.11	0.88	-	-	6.02	1.84	-	10.62	1	1

Table S2. Difference between recipient T-cell levels and vasoplegia

	Vasoplegia				No Vasoplegia				p value	q value
	Median	IQR (25-75)			Median	IQR (25-75)				
Treg CD4+	33.10	27.33	-	62.20	27.20	24.13	-	36.28	0.316	0.994
Treg CD4+ FOXP3+	4.57	2.48	-	6.89	8.18	3.79	-	17.53	0.133	0.994
Treg CD4+ FOXP3+ CCR5+	94.25	63.58	-	98.60	84.05	74.51	-	97.70	0.599	0.994
Treg CD4+ FOXP3+ CCR5+ Q1: CCR4- , CXCR3+	0	0	-	0	0	0	-	0.14	0.379	0.994
Treg CD4+ FOXP3+ CCR5+ Q2: CCR4+ , CXCR3+	39.55	7.42	-	66.75	14.95	4.58	-	34.30	0.521	0.994
Treg CD4+ FOXP3+ CCR5+ Q3: CCR4+ , CXCR3-	60.20	31.83	-	86.25	63.10	51.78	-	80.90	0.862	0.994
Treg CD4+ FOXP3+ CCR5+ Q4: CCR4- , CXCR3-	1.02	0.21	-	6.75	3.68	0.24	-	35.60	0.599	0.994
Treg CD4+ FOXP3+ CCR5-	5.73	1.42	-	36.42	15.95	2.28	-	25.48	0.684	0.994
Treg CD4+ FOXP3+ CD45RA+	7.44	6.54	-	28.31	8.42	3.07	-	19.63	0.77	0.994
Trm CD8+	24.80	18.65	-	31.55	22.90	15.90	-	32.35	0.703	0.994
Trm TCD8+/CCR5+ CXCR3+	7.70	2.29	-	50.08	5.84	2.81	-	38.45	1	1
Trm CD8+/CD69+ CD103+	16.00	10.55	-	22.63	17.30	12.15	-	30.85	0.549	0.994
Trm CD8+/CD69+ CD103+/CCR5+ CXCR3+	6.29	1.53	-	69.17	4.77	2.10	-	46.35	0.956	0.999
Trm CD8+/CD69+ CD103+/CD49a+	40.45	31.70	-	55.95	44.60	28.95	-	66.25	0.785	0.994
Tex CD4+	32.35	23.93	-	53.08	26.00	20.55	-	36.05	0.477	0.994
Tex CD4+/CCR5+	89.85	77.85	-	95.70	79.10	36.55	-	90.55	0.35	0.994
Tex CD4+/CCR5+/CXCR3+	28.05	11.93	-	74.70	20.20	14.30	-	30.95	0.785	0.994
Tex CD4+/CCR5+/CXCR3+/PD-1+	44.50	20.28	-	63.25	37.10	11.00	-	63.95	0.785	0.994
Tex CD4+ /CCR5+/CXCR3+/PD-1+/TIM-3+	6.70	4.27	-	16.37	5.06	1.85	-	9.055	0.296	0.994
Tex CD4+ /CCR5+/CXCR3+/TIM-3+	7.43	3.52	-	13.93	6.13	2.76	-	11.64	0.624	0.994
Tex CD4+ /CCR5-	10.18	4.31	-	22.15	20.90	9.44	-	63.45	0.35	0.994
Tex CD4+ /CCR5-/CXCR3-	48.35	35.25	-	86.95	70.30	48.25	-	94.10	0.477	0.994
Tex CD4+ /CCR5-/CXCR3-/PD-1+	18.31	8.55	-	74.4	8.07	4.25	-	23.95	0.202	0.994
Tex CD4+/CCR5-/CXCR3-/TIM-3+	4.38	2.17	-	7.94	1.62	0.74	-	6.23	0.296	0.994
Tex CD8+	25.35	18.60	-	31.50	26.10	15.80	-	32.50	1	1
TexCD8+/CCR5+	95.80	89.85	-	98.60	95.40	74.10	-	98.15	0.624	0.994
Tex CD8+/CCR5+/CXCR3+	19.81	8.91	-	79.88	14.60	8.49	-	26.10	0.785	0.994
Tex CD8+/CCR5+/CXCR3+/PD-1+	46.60	18.23	-	71.08	46.50	7.81	-	65.55	0.549	0.994
Tex CD8+/CCR5+/CXCR3+/PD-1+/TIM-3+	2.42	1.47	-	3.62	2.64	1.07	-	4.75	0.956	0.994
Tex CD8+/CCR5+/CXCR3+/TIM-3+	1.46	0.88	-	2.95	1.41	0.71	-	2.98	0.785	0.994

Tex CD8+/CCR5-	4.25	1.41 - 10.16	4.57	1.86 - 25.90	0.624	0.994
TexCD8+/CCR5-/CXCR3-	27.75	19.03 - 82.23	96.20	34.15 - 97.15	0.35	0.994
Tex CD8+/CCR5-/CXCR3-/PD-1+	7.40	3.90 - 28.78	2.60	1.92 - 6.47	0.163	0.994
TexCD8+/CCR5-/CXCR3-/TIM-3+	11.38	8.58 - 23.70	3.76	1.09 - 7.27	0.032	0.810
